# Impact of unstable housing on all-cause mortality among persons who inject drugs

**DOI:** 10.1186/s12889-015-1479-x

**Published:** 2015-02-07

**Authors:** Rebecca Zivanovic, MJ Milloy, Kanna Hayashi, Huiru Dong, Christy Sutherland, Thomas Kerr, Evan Wood

**Affiliations:** British Columbia Centre for Excellence in HIV/AIDS, St Paul’s Hospital, 608-1081 Burrard St, Vancouver, BC V6Z 1Y6 Canada; Department of Medicine, University of British Columbia, Vancouver, Canada

**Keywords:** Unstable housing, Injection drug use, Mortality

## Abstract

**Background:**

Illicit drug injecting is a well-established risk factor for morbidity and mortality. However, a limited number of prospective studies have examined the independent effect of unstable housing on mortality among persons who inject drugs (PWIDs). In this study we sought to identify if a relationship exists between unstable housing and all-cause mortality among PWIDs living in Vancouver, Canada.

**Methods:**

PWIDs participating in two prospective cohort studies in Vancouver, Canada were followed between May 1996 and December 2012. Cohort data were linked to the provincial vital statistics database to ascertain mortality rates and causes of death. We used multivariate Cox proportional hazards regression to determine factors associated with all-cause mortality and to investigate the independent relationship between unstable housing and time to all-cause mortality.

**Results:**

During the study period, 2453 individuals were followed for a median of 69 months (Inter-quartile range [IQR]: 34 – 113). In total, there were 515 (21.0%) deaths for an incidence density of 3.1 (95% Confidence Interval [CI]: 2.8 – 3.4) deaths per 100 person years. In multivariate analyses, after adjusting for potential confounders including HIV infection and drug use patterns, unstable housing remained independently associated with all-cause mortality (adjusted hazard ratio [AHR] = 1.30, 95% CI: 1.08 – 1.56).

**Conclusions:**

These findings demonstrate that unstable housing is an important risk factor for mortality independent of known risk factors including HIV infection and patterns of drug use. This study highlights the urgent need to provide supportive housing interventions to address elevated levels of preventable mortality among this population.

## Background

The link between high-intensity illicit drug use and elevated rates of preventable morbidity and mortality has been well documented. Furthermore, rates of illicit drug use and drug–related deaths have generally increased internationally in many settings over the last two decades [1]. Understanding the factors which increase the likelihood of premature mortality among persons who inject drugs (PWID) could inform the development of effective programs to improve both individual and community health.

The criminalization of people who use illicit drugs makes accurate study of the prevalence of illicit drug use and health-related outcomes, including mortality, challenging [2]. Nevertheless, mortality rates in studies of opioid users have been reported between six and 30 times that of age- and gender- matched non-opioid using individuals [3,4]. Similarly, mortality rates in cocaine users have been reported between four and six times that of age- and gender- matched peers [5]. Premature mortality in PWID is the result of a variety of causes including both acute and chronic diseases, accidents, violence and accidental overdose [3,6,7]. A recent systematic review and meta-analysis of mortality in PWID showed the two most common causes of death to be AIDS and overdose [6].

Homelessness and unstable housing often co-occur with illicit drug use [8,9]. Drug-use disorders independently increase the risk of first-time homelessness, suggesting prevention and treatment of substance use disorders could have a positive effect on homelessness [10]. In addition, homelessness has been linked with subsequent increases in drug use, injection-related risk behaviour and relapse in those who have stopped injecting drugs suggesting that effective interventions to promote housing have the potential to decrease the negative effects of drug use [11,12].

The role of homelessness as a risk factor for mortality has been well studied [13,14]. Large cohort studies in the United States show age-adjusted mortality among homeless persons to be three to nine times that of the general population [13,14]. Even after removing those with substance abuse from the group the difference remained three times greater than the general population [13]. A study conducted in Toronto showed that homeless women between the ages of 18 to 44 were 10 times more likely to die than women in the general population [15]. More recently, homelessness was shown to be an independent risk factor for mortality after adjustment for socioeconomic status and morbidity [16].

While studies have described the impacts of drug use patterns and comorbidities like HIV infection on mortality among persons who use drugs [17], few studies have been able to examine the effects of unstable housing on mortality after adjustment for potential confounders like prospective measures of drug use patterns and HIV infection especially in settings with universal health care systems where HIV treatment and care is provided free of charge. Therefore, the present study was conducted to examine the independent relationship, if any, between unstable housing and all-cause mortality among PWID living in Vancouver, Canada.

## Methods

The Vancouver Injection Drug Users Study (VIDUS) and AIDS Care Cohort to Evaluate Access to Survival Services (ACCESS) are open prospective cohorts of illicit drug users in Vancouver. The recruitment and follow up procedures for the two studies are largely identical to allow for analyses of merged data, with the only key differences being that HIV positive individuals are followed in ACCESS whereas HIV-negative individuals are followed in VIDUS. In both studies the primary modes of enrollment were self-referral, word of mouth, and street outreach. Detailed sampling and recruitment procedures for these two cohorts have been described elsewhere [18,19].

To be eligible, participants were 18 years of age or older, had ever injected illicit drugs and resided in the greater Vancouver region. Participants with no completed follow up surveys were excluded. All participants provided written informed consent. Participants were given a $20 stipend at each study visit for their time and transportation. The study was approved by the University of British Columbia/Providence Healthcare Research Ethics Board.

At baseline and semianually thereafter, participants completed an interviewer-administered questionnaire that elicited a range of data, including demographic characteristics, housing status, injection and non-injection drug use, and sexual risk behaviors. In addition, venous blood samples were drawn at each visit and tested for HIV and hepatitis C virus (HCV) antibodies among those previously testing negative for these diseases. Venous blood samples taken from HIV positive individuals were assessed for HIV disease progression [18]. All participants had private interviews and were offered both pre- and post-test counseling with trained nurses. Referral for free healthcare was provided to those who tested HIV positive and these individuals were subsequently followed in ACCESS.

The present study included PWID who were recruited and completed at least one follow up visit between May 1996 and December 2012. To avoid potential bias relating to long durations between the last study visit where behavioural information was assessed and the date of death, individuals who were identified as deceased more than 24 months after their last follow up visit were censored on the date of the last follow up.

The primary endpoint in this analysis was all-cause mortality. Here, we ascertained all-cause mortality rates among participants through a confidential record linkage using personal health numbers with the British Columbia Vital Statistics Agency and through ongoing follow up with contacts provided by participants. The primary explanatory variable of interest was unstable housing in the previous six months. As previously, unstable housing was defined as living in a single room occupancy hotel, shelter or other transitional housing, or living on the street [20,21].

Potential confounders that were considered included gender (male vs. female); age (per year older); ancestry (Caucasian vs. non-Caucasian); HIV serostatus (positive vs. negative); and sex work involvement (yes vs. no). A number of substance use behaviors (in the previous six months) were also considered, including ≥ daily heroin injection (yes vs. no), ≥ daily cocaine injection (yes vs. no), ≥ daily crack cocaine smoking (yes vs. no), and current enrolment in a methadone program (yes vs. no). Other covariates that were considered included incarceration (yes vs. no) and HCV serostatus (positive vs. negative). With the exception of age, gender and ancestry, all variables were in reference to the prior six months and measured at each semiannual follow up visit and were treated as time-updated.

As a first step, we used Chi-square test and Wilcoxon rank sum test to compare the baseline characteristics of the participants who did and did not report unstable housing at baseline. Those who did not report unstable housing at baseline were maintained as the reference group. All-cause mortality rate and 95% confidence interval [CI] were calculated using the Poisson distribution. Survival probabilities from all-cause mortality were estimated using the Kaplan-Meier product limit method, and compared using the two-sample log-rank test.

Next, we used bivariate Cox proportional hazards regression to examine the associations between each explanatory variable and time to all-cause mortality. To fit the multivariate model, we employed a conservative stepwise backward selection approach which considered the magnitude of change in the coefficient of unstable housing [22]. Specifically, we included all variables found to be associated with time to all-cause mortality in bivariate analyses at *p* < 0.10 in a multivariate model and used a stepwise approach to fit a series of reduced models. After comparing the value of the coefficient associated with unstable housing in the full model to the value of the coefficient in each of the reduced models, we dropped the secondary variable associated with the smallest relative change. We continued this iterative process until the minimum change exceeded 5%. Remaining variables were considered as potential confounders in a final multivariate model. All statistical analyses were performed using SAS software version 9.3 (SAS, Cary, NC). All *p*-values were two-sided.

## Results

A total of 2742 eligible individuals were recruited between May 1996 and December 2012. Two hundred eighty-nine participants were excluded based on the eligibility criteria described above leaving a sample of 2453 (89.5%) for further analysis. These individuals were followed for a median of 69 months (Inter-quartile range [IQR]: 34 to 113 months). Compared to the analytic sample, those not eligible were younger, less likely to be HCV seropositive, and less likely to use crack cocaine (all *p* <0.05), but there was no difference by housing status (*p* = 0.338). Among the study sample, 515 (21.0%) individuals died for an incidence density of mortality of 3.1 (95% CI: 2.8 – 3.4) deaths per 100 person years.

At baseline, 1602 participants (66.0%) were men, 758 (30.9%) were HIV positive, 2025 (82.6%) were HCV antibody positive and 1496 (61.0%) reported Caucasian ancestry. The median age was 37.7 years (IQR: 29.7-44.1). Five hundred sixty (22.8%) had histories of sex work and 326 (13.3%) had ever been incarcerated. At baseline, 909 (37.1%) of the study sample injected heroin at least daily in the previous six months, 733 (29.9%) injected cocaine at least daily, 590 (24.1%) smoked crack cocaine daily, and 559 (22.8%) were enrolled in a methadone program.

Table 1 reports the baseline characteristics of the study participants stratified by unstable housing. Compared to those with stable housing, those who reported unstable housing were more likely to be HIV seropositive (odds ratio [OR] = 1.23, 95% CI: 1.02 – 1.50) and to be HCV seropositive (OR = 1.59, 95% CI: 1.27-1.98). They were also more likely to have been involved in sex work (OR = 1.27, 95% CI: 1.03-1.58) and have been incarcerated (OR = 1.71, 95% CI: 1.29-2.28). They were less likely to be enrolled in a methadone program (OR = 0.82, 95% CI: 0.67 – 1.00). In terms of drug use patterns, unstable housing was statistically significant and positively associated with daily heroin injection (OR = 1.24, 95% CI: 1.04-1.49), daily cocaine injection (OR = 1.65, 95% CI: 1.35 – 2.02) and daily crack smoking (OR = 2.87, 95% CI: 2.25 – 3.66).

**Table 1:** **Baseline characteristics of the study participants stratified by unstable housing at baseline (n = 2453)** ^**1**^

		**Unstable housing**			
**Characteristic**	**Total n (%)**	**Yes 1713 (69.8)**	**No** ^**2**^ **722 (29.4)**	**Odds ratio (95% CI)**	***p*** **-value**
**Gender**					
**Male**	1620 (66.0)	1147 (67.0)	462 (64.0)	1.14 (0.95-1.37)	0.158
**Female**	833 (34.0)	566 (33.0)	260 (36.0)		
**Age, in years (median IQR)**	37.7 (29.7 – 44.1)	37.8 (30.0 – 44.1)	37.6 (29.6 – 44.0)	1.00 (0.99-1.01)	0.399
**Ancestry**					
**Caucasian**	1496 (61.0)	1058 (61.8)	430 (59.6)	1.10 (0.92-1.31)	0.308
**Other**	957 (39.0)	655 (38.2)	292 (40.4)		
**HIV serostatus**					
**Positive**	758 (30.9)	551 (32.2)	200 (27.7)	1.23 (1.02-1.50)	0.032
**Negative**	1692 (69.0)	1161 (67.8)	520 (72.0)		
**Sex work involvement** ^**3**^					
**Yes**	560 (22.8)	410 (23.9)	143 (19.8)	1.27 (1.03-1.58)	0.026
**No**	1883 (76.8)	1296 (75.7)	576 (79.8)		
**Daily heroin injection** ^**3**^					
**Yes**	909 (37.1)	660 (38.5)	242 (33.5)	1.24 (1.04-1.49)	0.020
**No**	1538 (62.7)	1049 (61.2)	478 (66.2)		
**Daily cocaine injection** ^**3**^					
**Yes**	733 (29.9)	560 (32.7)	164 (22.7)	1.65 (1.35-2.02)	< 0.001
**No**	1704 (69.5)	1143 (66.7)	552 (76.5)		
**Daily non-injection cocaine use** ^**3**^					
**Yes**	590 (24.1)	496 (29.0)	90 (12.5)	2.87 (2.25-3.66)	< 0.001
**No**	1860 (75.8)	1214 (70.9)	632 (87.5)		
**Enrolment in a methadone program** ^**3**^					
**Yes**	559 (22.8)	373 (21.8)	184 (25.5)	0.82 (0.67-1.00)	0.051
**No**	1885 (76.8)	1332 (77.8)	537 (74.4)		
**Incarceration** ^**3**^					
**Yes**	326 (13.3)	255 (14.9)	67 (9.3)	1.71 (1.29-2.28)	<0.001
**No**	2122 (86.5)	1454 (84.9)	654 (90.6)		
**HCV serostatus**					
**Positive**	2025 (82.6)	1450 (84.7)	562 (77.8)	1.59 (1.27-1.98)	<0.001
**Negative**	411 (16.8)	252 (14.7)	155 (21.5)		

1. Not all cells add up to 2453 as participants may choose not to answer sensitive questions.

2. The reference group is those who do not report unstable housing at baseline.

3. Refers to behaviors in the last six months.

IQR = inter-quartile range.

**Figure 1:**
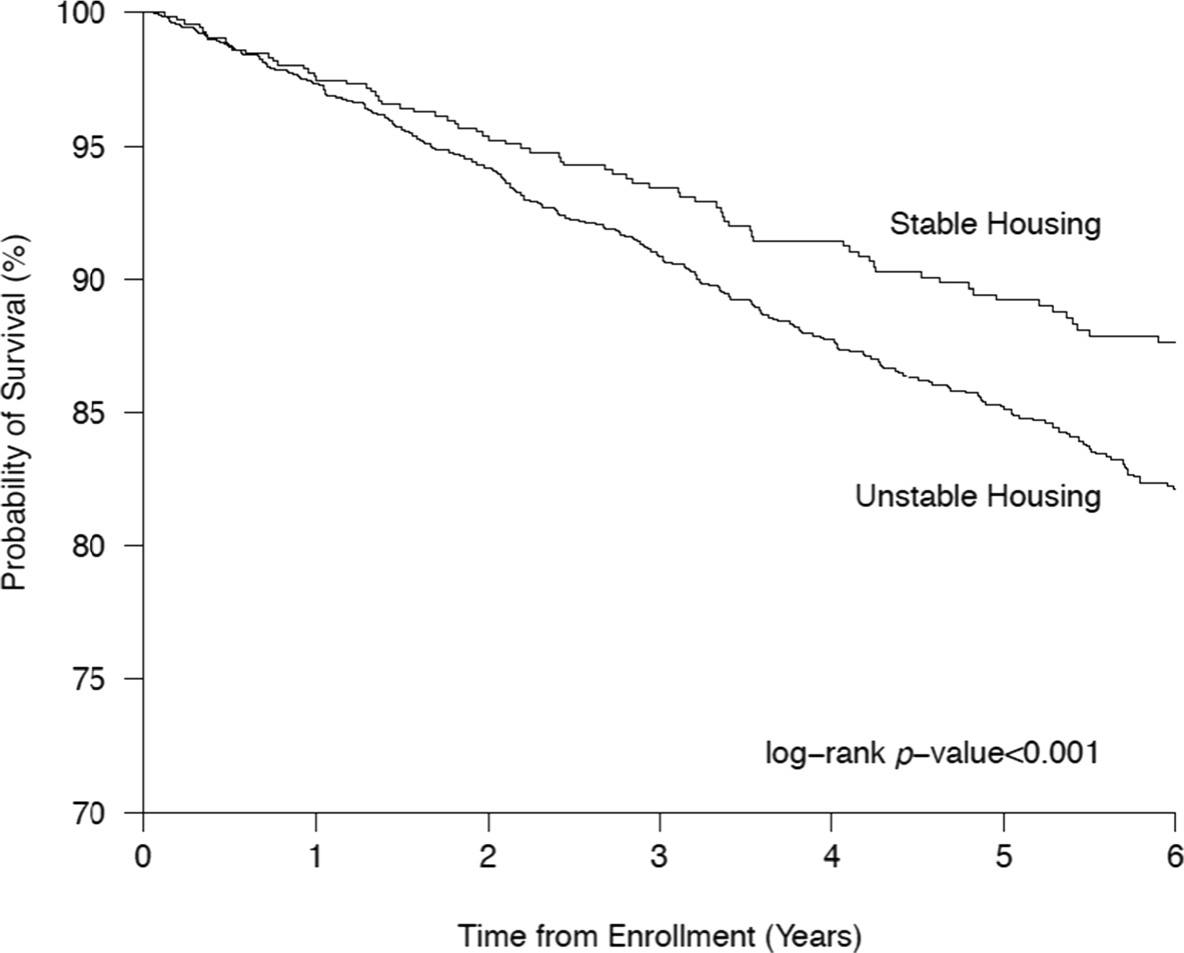
**Probability of survival for PWID with stable housing vs those with unstable housing.**

Figure 1 shows the results of the Kaplan-Meier analysis of time to all-cause mortality stratified by baseline housing status. As shown, individuals reporting unstable housing were significantly more likely to die during follow up than individuals reporting stable housing at baseline (*p* < 0.001).

Table 2 shows results of the bivariate and multivariate Cox regression analyses of time to all-cause mortality. In the bivariate analysis, unstable housing was statistically significant and positively associated with time to all-cause mortality with a relative hazard (RH) of 1.37 (95% CI: 1.14-1.63).

**Table 2:** **Bivariate and multivariate Cox proportional hazards analyses of the time to death among 2453 PWID**

	**Unadjusted**			**Adjusted**		
	**Relative hazard (RH)**			**Relative hazard (ARH)**		
**Variable**	**RH**	**(95% CI)**	***p*** **-value**	**ARH**	**(95% CI)**	***p*** **-value**
Unstable housing*						
(Yes vs. No)	1.37	1.14-1.63	<0.001	1.30	1.08-1.56	0.005
**Gender**						
(Male vs. Female)	1.09	0.91-1.30	0.353			
**Age**						
(per 1 year older)	1.03	1.02-1.04	<0.001			
**Ethnicity**						
(Caucasian vs. Other)	1.07	0.90-1.28	0.440			
**HIV serostatus**						
(Positive vs. Negative)	2.67	2.24-3.18	<0.001	2.56	2.14-3.07	<0.001
Sex work involvement*						
(Yes vs. No)	0.76	0.57-1.02	0.071			
Daily heroin injection*						
(Yes vs. No)	0.80	0.64-1.00	0.051	0.83	0.66-1.04	0.108
Daily cocaine injection*						
(Yes vs. No)	1.44	1.16-1.80	0.001	1.36	1.08-1.71	0.009
Daily Non-injection cocaine use*						
(Yes vs. No)	0.89	0.73-1.08	0.238			
Enrolment in a methadone program*						
(Yes vs. No)	0.81	0.68-0.97	0.020			
Incarceration*						
(Yes vs. No)	0.92	0.72-1.17	0.486			
**HCV serostatus**						
(Positive vs. Negative)	1.95	1.30-2.94	0.001			

*Behaviours refer to activities in the last six months.

As also shown in Table 2, in multivariate analyses, after adjusting for potential confounders including HIV infection and drug use patterns, unstable housing remained independently associated with all-cause mortality (adjusted hazard ratio [AHR] = 1.30, 95% CI: 1.08 – 1.56).

## Discussion

The aim of this study was to investigate the relationship between unstable housing and risk of death among people who inject drugs. We demonstrated that among a large cohort of PWID, unstable housing was independently associated with all-cause mortality. This association persisted after adjustment for a range of prospectively measured potential confounding variables including high-intensity (greater than or equal to daily) drug-use behaviours and HIV serostatus.

When stratified by housing status the baseline characteristics of the two groups showed that those in the unstable housing group were more likely to be HCV seropositive, HIV seropositive, involved in sex work or to have been incarcerated. These individuals with unstable housing were also less likely to be involved in a methadone program and more likely to report at least daily heroin injection, at least daily cocaine injection and at least daily crack smoking. These characteristics illustrate specific examples of the daily challenges faced by those living with unstable housing compared to their housed counterparts. These social, behavioural and medical factors associated with the group of PWID reporting unstable housing also attests to the complexity of both living as and studying this population.

Interestingly, even after adjustment for these competing risks of death, our analyses demonstrated a positive association between unstable housing and mortality compared to their housed counterparts suggesting that, despite all of the factors at play in the challenging lives of PWID, housing stability alone can have a substantial impact on survival.

Our findings of increased mortality in PWID with unstable housing are consistent with the available literature supporting increased mortality in homeless populations in general [13,15]. Specifically, the present research adds important data on the subset of homeless individuals who also use drugs and demonstrates the important role of housing on survival. Our data supports existing literature highlighting the importance of framing homelessness as a health issue, and creating public health interventions aimed at improving the health and longevity of drug users by addressing various social and environmental factors, including homelessness, that worsen the negative consequences of drug use [23].

Housing is included with food, clothing, medical care and necessary social services under Article 25 of the United Nations Declaration of Human Rights [24]. However provision lags behind and often comes with restrictions and stipulations around sobriety, health status, employment and income. One specific example of a novel approach at work is “Housing First” which involves providing access to permanent housing and the services people may desire to maintain that housing. This program works on the philosophy shared by the UN that housing is a basic human right and then the many factors that may have contributed to a person’s homelessness can be best addressed once the person has stable housing [25].

Our study has several limitations. First, the study sample was not randomly selected and our findings may not be generalizable to all PWID. Second, although the self-reported data may have been affected by reporting bias, we do not believe that individuals could have differentially reported housing status based on their time to all-cause-mortality, which was ascertained through administrative data. Self-reported data to control for potential confounding has been commonly used and found to be valid in studies involving PWID [26]. Third, as with all observational studies, the relationship observed between mortality and unstable housing may be under the influence of unobserved confounding. Finally, mortality rates may have been underestimated as participants who died outside of the province were not included in the provincial registry and therefore not accounted for. However, this is unlikely to impact our findings as previous studies have shown that migration rates out of province are very low in this setting [27].

## Conclusions

In summary, the present study highlights the significant role of unstable housing in increasing premature mortality in PWID. This demonstrates the need for implementation of supportive housing programs to be an integral part of public health initiatives aimed at improving the health of populations of PWID.

## Abbreviations

ACCESS: AIDS Care Cohort to Evaluate Access to Survival Services
AHR: Adjusted Hazard Ratio
AIDS: Acquired Immunodeficiency Syndrome
CI: Confidence Interval
HCV: Hepatitis C Virus
HIV: Human Immunideficiency Virus
IQR: Inter-quartile range
PWID: Persons who inject drugs
OR: Odds Ratio
UN: United Nations
VIDUS: Vancouver Injection Drug Users Study
